# Supplementary material for: Seroprevalence, Waning and Correlates of Anti-SARS-CoV-2 IgG Antibodies in Tyrol, Austria: Large-Scale Study of 35,193 Blood Donors Conducted between June 2020 and September 2021
Source: Viruses. 2022 Mar 9;14(3):568. doi: 10.3390/v14030568 (PMC8954543; doi:10.3390/v14030568)
Supplement: Supplementary file 1 [file viruses-14-00568-s001.zip › viruses-1609045-supplementary.pdf]

Supplementary Material

Supplementary Tables

Table S1. STROBE checklist.

	Item No	Recommendation	Page No
Title and abstract	1	(*a*) Indicate the study's design with a commonly used term in the title or the abstract	1
		(*b*) Provide in the abstract an informative and balanced summary of what was done and what was found	1
**Introduction**			
Background/ rationale	2	Explain the scientific background and rationale for the investigation being reported	1-2
Objectives	3	State specific objectives, including any prespecified hypotheses	2
**Methods**			
Study design	4	Present key elements of study design early in the paper	2-3
Setting	5	Describe the setting, locations, and relevant dates, including periods of recruitment, exposure, follow-up, and data collection	2-3
Participants	6	(*a*) Give the eligibility criteria, and the sources and methods of selection of participants. Describe methods of follow-up	2
		(*b*) For matched studies, give matching criteria and number of exposed and unexposed	NA
Variables	7	Clearly define all outcomes, exposures, predictors, potential confounders, and effect modifiers. Give diagnostic criteria, if applicable	2-3
Data sources/ measurement	8*	For each variable of interest, give sources of data and details of methods of assessment (measurement). Describe comparability of assessment methods if there is more than one group	2-3
Bias	9	Describe any efforts to address potential sources of bias	3
Study size	10	Explain how the study size was arrived at	2-3
Quantitative variables	11	Explain how quantitative variables were handled in the analyses. If applicable, describe which groupings were chosen and why	3
Statistical methods	12	(*a*) Describe all statistical methods, including those used to control for confounding	3
		(*b*) Describe any methods used to examine subgroups and interactions	3
		(*c*) Explain how missing data were addressed	3
		(*d*) If applicable, explain how loss to follow-up was addressed	NA
		(*e*) Describe any sensitivity analyses	3
**Results**			
Participants	13*	(a) Report numbers of individuals at each stage of study—eg numbers potentially eligible, examined for eligibility, confirmed eligible, included in the study, completing follow-up, and analyzed	3-9
		(b) Give reasons for non-participation at each stage	3-9
		(c) Consider use of a flow diagram	NA
Descriptive data	14*	(a) Give characteristics of study participants (eg demographic, clinical, social) and information on exposures and potential confounders	3, Table 1
		(b) Indicate number of participants with missing data for each variable	Table 1

		of interest<br>(c) Summarize follow-up time (eg, average and total amount)	Table 1
Outcome data	15*	Report numbers of outcome events or summary measures over time	4-9
Main results	16	(a) Give unadjusted estimates and, if applicable, confounder-adjusted estimates and their precision (eg, 95% confidence interval). Make clear which confounders were adjusted for and why they were included	4-9
		(b) Report category boundaries when continuous variables were categorized	4-9
		(c) If relevant, consider translating estimates of relative risk into absolute risk for a meaningful time period	NA
Other analyses	17	Report other analyses done—eg analyses of subgroups and interactions, and sensitivity analyses	5-9
**Discussion**			
Key results	18	Summarize key results with reference to study objectives	9-10
Limitations	19	Discuss limitations of the study, taking into account sources of potential bias or imprecision. Discuss both direction and magnitude of any potential bias	11
Interpretation	20	Give a cautious overall interpretation of results considering objectives, limitations, multiplicity of analyses, results from similar studies, and other relevant evidence	10-11
Generalizability	21	Discuss the generalizability (external validity) of the study results	10-11
**Other information**			
Funding	22	Give the source of funding and the role of the funders for the present study and, if applicable, for the original study on which the present article is based	12

Table S2. Seroprevalences in all participants (principal analysis), in participants with repeated donations since October 2017, and in all participants with age- and sex-standardisation across the total population of Tyrol.

Month	% seropositive (95% CI)		
	All participants (n=35,193)	Participants who had already donated blood leading up to the study[a] (n=21,361)	All participants (n=35,193) with age- and sex-standardisation across the total population of Tyrol[b]
**Anti-N IgG**			
June 2020	3.4 (2.8-4.2)	2.5 (2.0-3.3)	3.3 (2.6-4.0)
July 2020	2.7 (2.1-3.4)	2.6 (1.9-3.4)	2.6 (2.0-3.3)
October 2020	3.4 (2.9-4.0)	3.1 (2.5-3.7)	3.3 (2.7-3.9)
November 2020	8.2 (7.3-9.1)	7.4 (6.4-8.5)	7.8 (6.9-8.7)
December 2020	11.6 (10.6- 12.7)	11.4 (10.1-12.9)	11.2 (10.1-12.3)
January 2021	17.1 (16.0-18.3)	16.9 (15.5-18.4)	16.8 (15.7-18.0)
February 2021	15.2 (14.0-16.5)	16.5 (14.9-18.2)	15.3 (14.0-16.7)
March 2021	14.0 (13.0-15.1)	14.1 (12.9-15.5)	14.3 (13.5-15.2)
**Anti-S IgG**			
March 2021	29.9 (27.5-32.4)	29.4 (26.5-32.5)	29.1 (26.6-31.6)
April 2021	35.2 (33.5-37.0)	36.3 (34.2-38.5)	35.6 (33.7-37.5)
Mai 2021	49.4 (47.6-51.3)	55.1 (52.8-57.3)	52.2 (50.4-54.1)
June 2021	68.3 (66.8-69.8)	73.5 (71.6-75.3)	68.5 (67.0-70.1)
July 2021	75.9 (74.5-77.2)	79.9 (78.3-81.5)	76.0 (74.6-77.4)
August 2021	82.6 (81.2-83.9)	84.9 (83.2-86.4)	82.7 (81.4-84.0)
September 2021	82.7 (81.4-83.8)	84.4 (82.8-85.8)	82.6 (81.3-83.8)

[a]To define this subgroup of the study population, the period from October 2017 to study baseline was considered. [b]Direct age- and sex standardisation was applied by using age (categories 18-30, >30-40, >40-50, >50-60, >60-70 years) and sex structured data of the population of the Federal State of Tyrol in Austria as standard population as of 1 January 2021 from the Statistik Austria. Seroprevalence estimates by age groups and sex are presented in Supplementary Figures.

Supplementary Figures

Figure S1. Symptoms and medical attendance of prior SARS-CoV-2 infections.

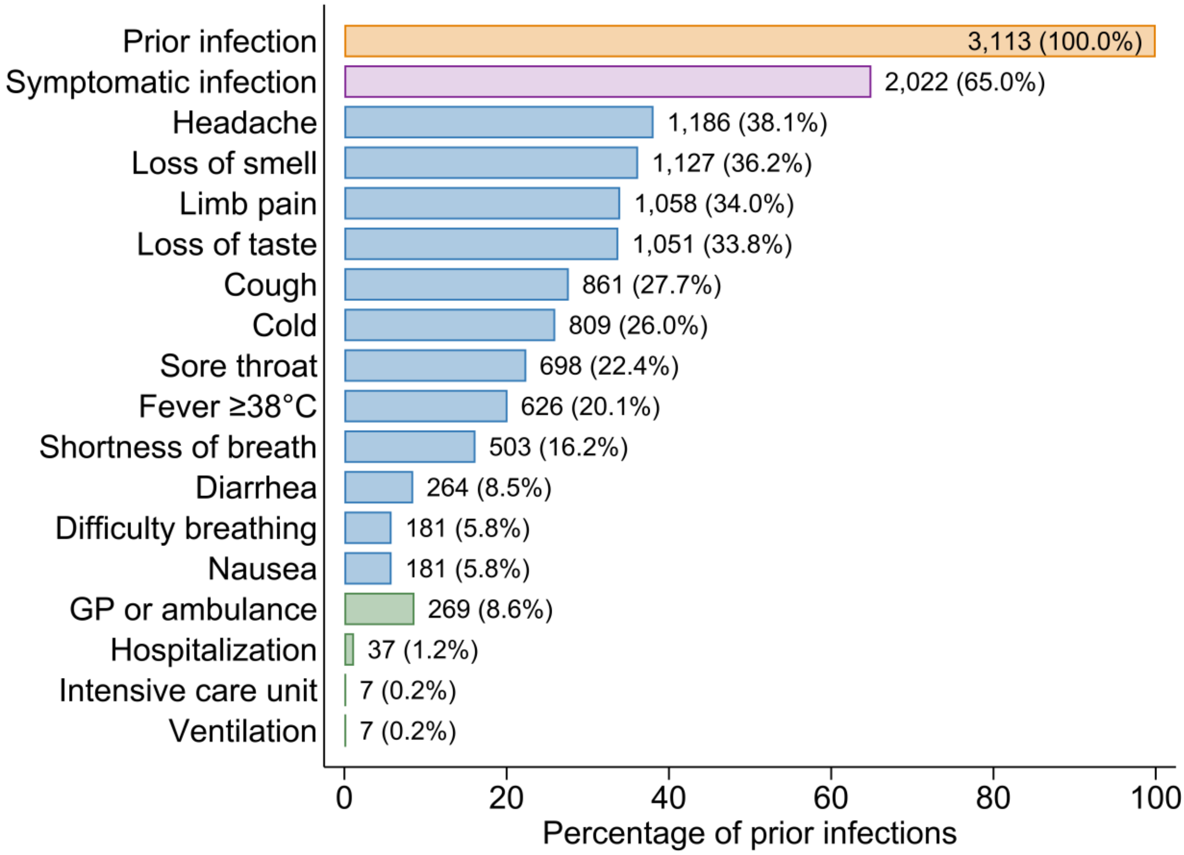

Abbreviations: GP, general practicer.

Figure S2. Seroprevalence of anti-SARS-CoV-2 IgG antibodies by sex and age group, Tyrol, Austria, June 2020-September 2021(n=35,193).

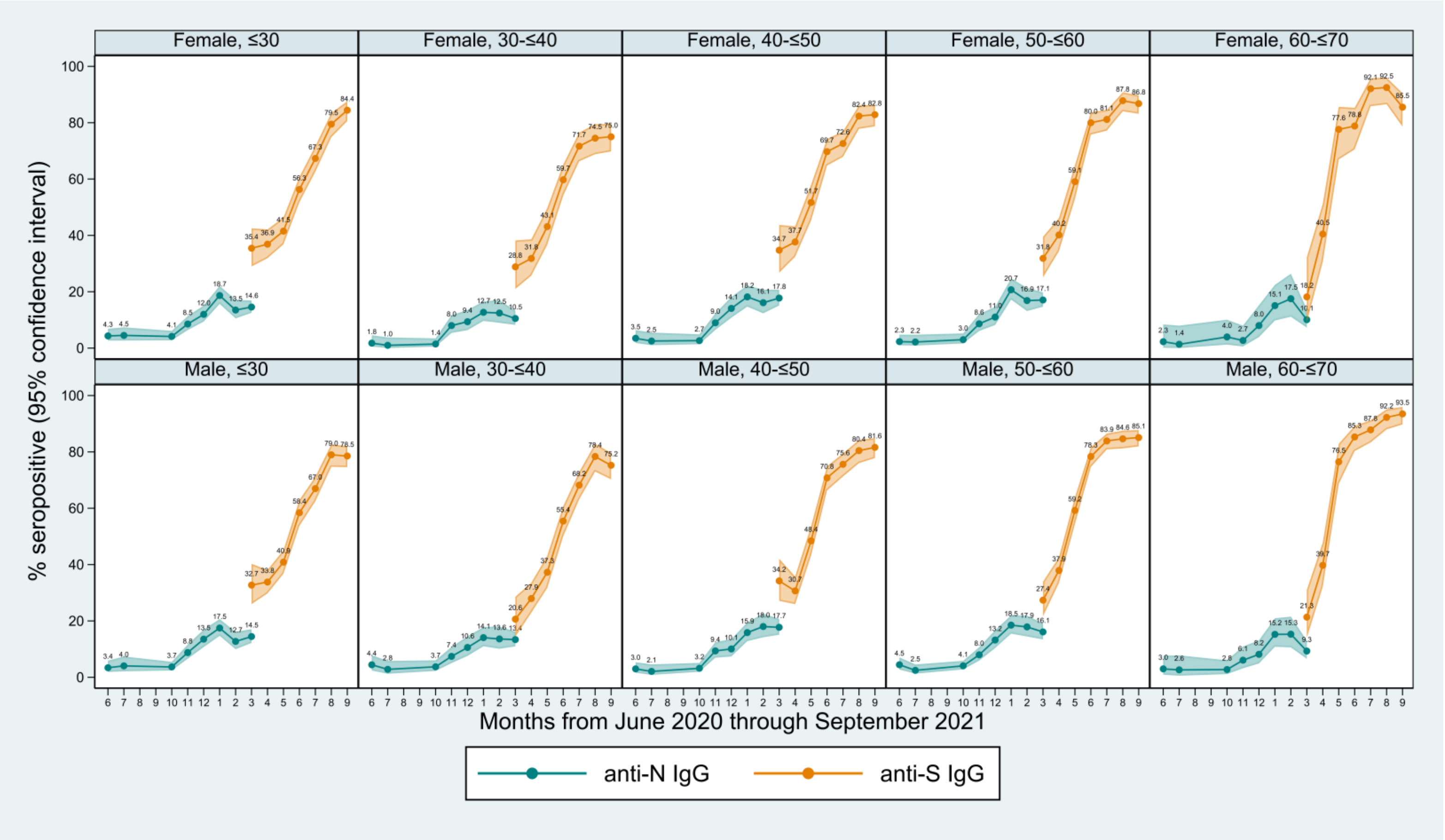

Figure S3. Seroprevalence of anti-S IgG antibodies among unvaccinated and of anti-N IgG antibodies for different time periods and across different population subgroups, Tyrol, Austria, June 2020-September 2021 (n=35,193).

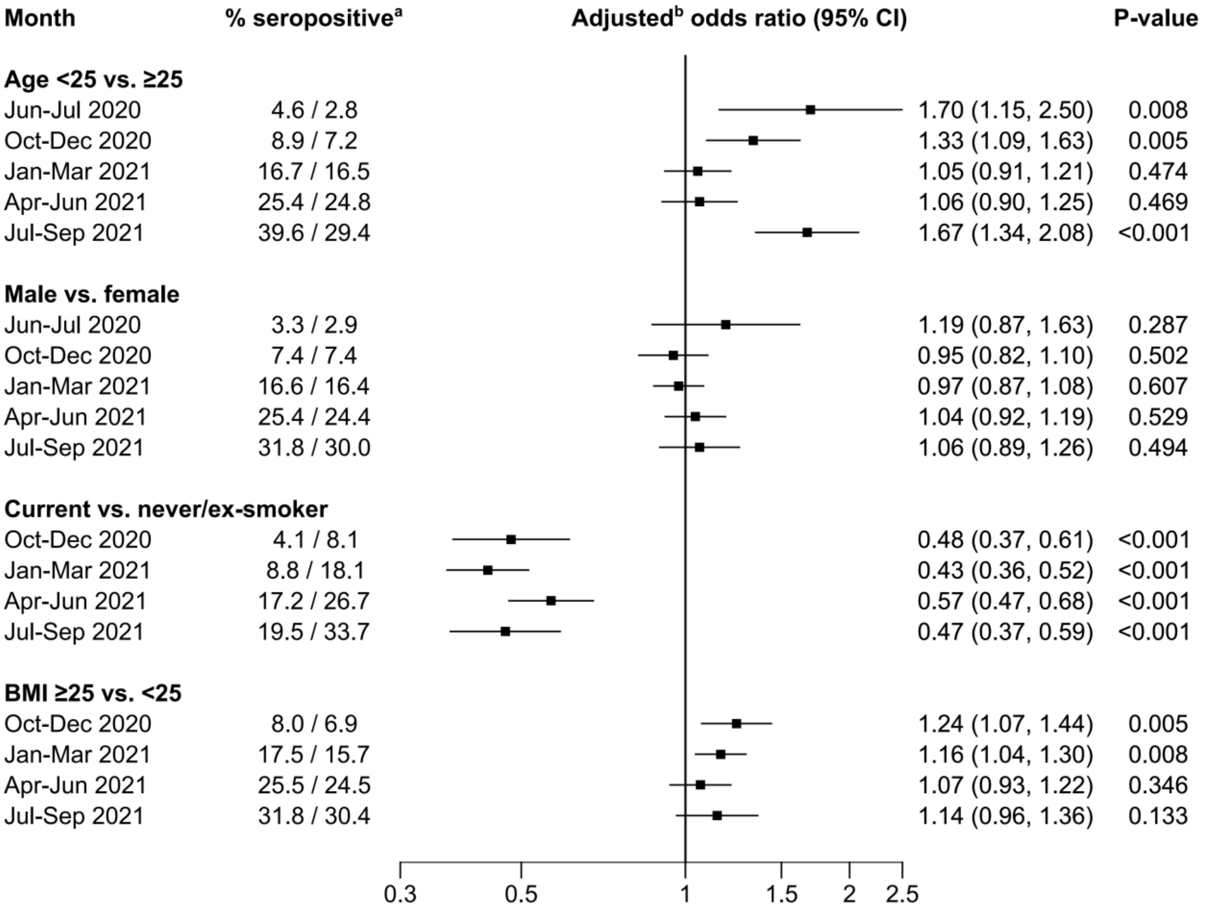

Abbreviations: BMI, body mass index; CI, confidence interval. [a]The reference group is depicted on the right side. [b]Adjusted for all the variables shown in this figure (age <25 vs. ≥25 years), male vs. female sex, current vs. never/ex-smoker, and body mass index (≥25 vs. <25 kg/m²)
